# Within-person reciprocal processes between AI feedback literacy and agentic engagement: a three-wave random-intercept cross-lagged panel study

**DOI:** 10.3389/fpsyg.2026.1893874

**Published:** 2026-10-22

**Authors:** Shuai Xu

**Affiliations:** School of Marxism, Sias University, Xinzheng, China

**Keywords:** agentic engagement, AI feedback literacy, higher education, random-intercept cross-lagged panel model, reciprocal effects, self-determination theory

## Abstract

Artificial intelligence (AI) feedback has become a pervasive feature of university learning, yet whether students’ capacity to engage productively with such feedback co-develops with their broader learner agency remains poorly understood. Drawing on student feedback literacy theory, self-determination theory as a supplementary motivational lens, and the mutualism principle, this study tested whether AI feedback literacy and agentic engagement reciprocally predict each other over time at the within-person level and exploratorily examined whether they share a stable trait-level association. A three-wave panel study of 480 Chinese undergraduates (*N* = 399 with complete data across all waves), with measurements at six-week intervals across one semester, was analyzed using a random-intercept cross-lagged panel model (RI-CLPM). At the between-person level, students who scored systematically higher in AI feedback literacy across the semester also scored systematically higher in agentic engagement, with a moderate and statistically significant trait-level correlation between the two random intercepts (*r* = 0.315, *p* < 0.001), supporting Hypothesis 1. At the within-person level, both cross-lagged pathways were positive and significant: deviations in AI feedback literacy predicted later deviations in agentic engagement (*β* = 0.222, *p* < 0.001), and the reverse pathway was likewise significant (*β* = 0.263, *p* < 0.001). A Wald test indicated that the two reciprocal pathways did not differ significantly in magnitude (Δ*B* = 0.002, *p* = 0.972), consistent with a symmetric mutualism. A model comparison confirmed that the RI-CLPM provided substantially better fit than a traditional cross-lagged panel model, with the RI-CLPM revealing substantially larger within-person reciprocal effects once stable trait-level covariation was properly isolated. The findings extend the mutualism principle from a coupling of person-internal constructs to a coupling between a person-internal competency and a person-environment behavioral interface. They also raise the possibility, in a Confucian Heritage Culture context where agentic engagement has historically been culturally muted, that AI feedback may offer a less culturally-loaded venue in which students can exercise agency. The study repositions AI feedback literacy from a static skill into a dynamic developmental resource that is constituted, in part, by the very engagement it enables.

## 1 Introduction

Whether students can shape their own learning trajectories has long been treated as a central question in educational psychology, and the rise of generative artificial intelligence has placed that question under renewed pressure. When learners receive feedback from a teacher or a peer, the social contract of feedback is well understood: feedback comes from a knowledgeable other who is, in principle, accountable for its quality. When feedback comes from an AI system, that contract is suspended. The feedback is abundant, immediate, and seemingly authoritative, yet it issues from a source that cannot be questioned about its intent. Whether students grow into more autonomous learners under such conditions, or whether they slide into a posture of passive reliance, depends in part on two psychological resources that this study takes as its focus: their capacity to engage productively with feedback, and their willingness to act upon it.

The first resource, student feedback literacy, denotes the appreciation, evaluation, and uptake skills that allow learners to make feedback work for their own development (Carless and Boud, 2018; Molloy et al., 2020). Recent scholarship has begun to extend this construct to AI-mediated contexts, defining AI feedback literacy as the constellation of competencies through which students appreciate the affordances of AI feedback, judge its trustworthiness, manage their affective responses to it, and translate it into action (Liu and Deris, 2025). The second resource, agentic engagement, refers to the proactive, constructive contribution students make to the flow of instruction by asking questions, expressing preferences, offering input, and reshaping the learning activity itself (Reeve and Tseng, 2011). Although both constructs are now firmly established within educational psychology, they have largely traveled in separate literatures, and the empirical evidence on their interrelations remains thin.

There are strong theoretical grounds to expect AI feedback literacy and agentic engagement to be reciprocally entangled rather than unidirectionally related. A student who can interpret and act on AI feedback is precisely the student for whom asking the AI a follow-up question, contesting a suggestion, or steering the dialogue feels rewarding rather than risky; this is the logic of competence-supported autonomy described by self-determination theory (Ryan and Deci, 2000; Vansteenkiste et al., 2020). Conversely, students who already engage agentically with their learning environments tend to encounter the more demanding feedback situations in which literacy is developed, and they tend to do so repeatedly (Winstone et al., 2017a). Whether such reciprocal dynamics actually unfold within individual students over time, however, has not been directly tested. Existing work on feedback and engagement has been overwhelmingly cross-sectional or has relied on the traditional cross-lagged panel model (CLPM), a design that conflates trait-like differences between students with state-like changes within them (Hamaker et al., 2015; Lucas, 2023).

The random-intercept cross-lagged panel model (RI-CLPM) addresses this conflation by partitioning the longitudinal covariance structure into a stable between-person component and a fluctuating within-person component (Hamaker et al., 2015; Mulder and Hamaker, 2021). When applied to questions of reciprocity, the RI-CLPM offers a sharper test: it asks whether students’ deviations from their own typical levels of one construct anticipate deviations in the other, net of the trait differences that would otherwise inflate or distort such estimates. Despite the rapid uptake of RI-CLPM in motivation and engagement research (Orth et al., 2024; Vu et al., 2022), it has scarcely been applied within feedback literacy scholarship. The mismatch between the methodological sophistication required by reciprocal questions and the analytic strategies currently in use leaves a notable inferential gap.

The present study addresses this gap by examining the dynamic interplay between AI feedback literacy and agentic engagement across three measurement waves in a sample of Chinese undergraduates. We make three contributions. Theoretically, we integrate student feedback literacy theory, self-determination theory, and the mutualism principle to articulate why AI feedback literacy and agentic engagement should co-develop, and we extend feedback literacy theory to the AI feedback context where the social contract of feedback differs fundamentally from traditional teacher feedback. Methodologically, we apply RI-CLPM to disentangle between- and within-person reciprocal effects, providing the first such analysis at the intersection of AI feedback and student agency. Practically, our findings reposition AI feedback literacy from a static competence to be trained into a dynamic developmental resource that grows in tandem with, and partly depends on, learners’ agentic engagement.

## 2 Theoretical framework and hypotheses

### 2.1 Student feedback literacy in the age of AI feedback

Student feedback literacy emerged as an organizing construct to address a persistent finding in higher education: that feedback, no matter how high in quality on the giver’s side, often fails to produce learning gains on the receiver’s side (Carless and Boud, 2018; Winstone et al., 2017b). Rather than locating the problem in feedback delivery, the feedback literacy framework relocates it within the learner, conceptualizing the productive use of feedback as a literacy that students develop over time. Carless and Boud (2018) define student feedback literacy as the understanding, capacities, and dispositions needed to make sense of information about performance and to use it for enhancing work or learning strategies. Subsequent elaborations have refined this definition into four dimensions: appreciating the value of feedback, making judgments about its quality, managing affective responses, and taking action (Molloy et al., 2020). This four-dimensional structure has become the dominant analytic lens in the feedback literacy literature.

Empirical work on student feedback literacy has accumulated rapidly. Carless (2019) traced longitudinal feedback uptake processes and described how feedback experiences spiral across assignments, with each cycle either consolidating or eroding subsequent literacy. Yan and Carless (2022) showed that self-assessment competence depends on feedback literacy and not the reverse, foregrounding its enabling role. Chong (2021) reframed feedback literacy from an ecological perspective, attending to the discipline-specific, relational, and temporal contexts that shape its development. Across these accounts, feedback literacy is consistently treated as a relational achievement rather than a stable trait, even though the bulk of empirical evidence has remained cross-sectional.

The arrival of generative AI tools, particularly large language models capable of producing detailed feedback on student writing and other artifacts, has unsettled core assumptions of the feedback literacy framework. Three assumptions are most directly implicated. First, feedback in the traditional framework is a social act, embedded in an asymmetric but accountable relationship between giver and receiver (Henderson et al., 2019). AI feedback is generated by a non-intentional system whose outputs cannot be questioned about motive or context. Second, traditional feedback is bounded in supply: students receive limited feedback episodes per course. AI feedback is virtually unbounded, raising new questions about how learners evaluate and prioritize abundance. Third, traditional feedback varies in quality with the expertise of the giver. AI feedback can be plausibly worded yet substantively unreliable, making the judgment dimension of feedback literacy more consequential than ever (Steiss et al., 2024).

Recent work has begun to extend feedback literacy to the AI context. Liu and Deris (2025) developed and validated the AI Feedback Literacy Scale, which adapts the four-dimensional structure to AI feedback while adding critical evaluation of AI outputs as a distinguishing competency. Wang and Wang (2025) proposed the APSE model of critical AI literacy in writing, foregrounding the appraisal, prompting, sense-making, and ethical-use processes that learners enact when working with AI feedback. Alwafi (2025) examined how AI-assisted peer review reshapes students’ AI feedback literacy alongside their motivation and self-esteem, while Guo et al. (2024) showed that an AI-supported approach to peer feedback improves both feedback quality and downstream writing performance. These studies map the contours of AI feedback literacy as a competence, but they have stopped short of examining how it co-develops with the broader psychological resources that determine whether students put feedback to work.

It is important to distinguish AI feedback literacy from the broader construct of general AI literacy. General AI literacy encompasses the knowledge, attitudes, and competencies needed to understand, interact with, and critically evaluate AI systems across diverse contexts. AI feedback literacy, by contrast, is domain-specific: it captures the competencies students deploy specifically in response to AI-generated feedback events—appraising the relevance and accuracy of feedback outputs, managing affective reactions, and translating appraisals into revised work. A student may possess high general AI literacy (e.g., understanding how large language models generate text) yet lack the evaluative capacities needed to judge whether a particular piece of AI feedback on their essay is substantively sound. Meta-analytic evidence on AI and learner autonomy also suggests that access to AI support is not sufficient in itself: across 32 studies, AI support strengthened cognitive, metacognitive, and motivational regulation more consistently than behavioral regulation, and its benefits depended on how well interventions were aligned with learner characteristics and instructional goals (Achuthan, 2025). The present study focuses on this narrower, feedback-specific construct because it is the capacity most directly implicated in the learning loop between receiving AI feedback and acting upon it.

### 2.2 Agentic engagement and its place in self-determination theory

Agentic engagement was introduced by Reeve and Tseng (2011) as a fourth aspect of student engagement, alongside behavioral, emotional, and cognitive engagement. It is defined as the constructive contribution students make to the flow of instruction, manifest in actions such as asking questions, expressing preferences, offering input, telling the teacher what one needs, and suggesting different ways to proceed. Crucially, agentic engagement is conceptualized as behavior rather than capacity: it is what students do, not what they are able to do. This distinguishes it from related constructs such as self-efficacy, which describes belief, or self-regulated learning strategies, which describe procedural knowledge (Panadero, 2017).

Agentic engagement is theoretically situated within self-determination theory, which holds that students thrive when their basic psychological needs for autonomy, competence, and relatedness are satisfied (Ryan and Deci, 2000; Vansteenkiste et al., 2020). When these needs are met, students adopt a more autonomous motivational stance, and one behavioral expression of that stance is the willingness to enter the learning activity as an active participant rather than a passive recipient (Cheon et al., 2019; Reeve, 2013). Research on agentic engagement has shown that it predicts learning outcomes above and beyond the other three engagement components, and that teachers can foster it through autonomy-supportive instructional practices (Reeve and Tseng, 2011; Reeve, 2013).

Within AI-mediated learning environments, agentic engagement takes on a sharper edge. Acting agentically toward a human teacher carries social risks that are absent with AI: there is no teacher to displease, no peer audience to read one’s questions as deficient. At the same time, the lack of a knowledgeable interlocutor means that the cognitive work of agentic engagement, such as formulating a productive follow-up question or steering a feedback dialogue toward what one actually needs, falls more squarely on the student. Darvishi et al. (2024) showed that AI assistance can either support or supplant student agency depending on how learners enact it, raising the question of which psychological resources enable productive enactment.

A further distinction bearing on the present argument concerns the difference between the availability of AI feedback and students’ actual uptake of that feedback. The proliferation of AI-generated feedback does not guarantee productive engagement; in a survey of nearly 7,000 students at four Australian universities, most students rated both generative AI feedback and teacher feedback as helpful, yet they judged teacher feedback to be more trustworthy (Henderson et al., 2026). Credibility judgments of this kind are part of what determines whether feedback is acted upon or set aside. The capacity to judge, interpret, and act on AI feedback—the core of AI feedback literacy as defined above—thus constitutes a critical mediating link between feedback availability and its downstream behavioral impact. Moreover, the delivery mechanism of AI assistance matters. In a randomized experiment on AI-assisted decision-making, participants who received assistance only on request engaged more critically with the advice and were less likely to be misled than participants who received unsolicited assistance (Nair et al., 2026). Assistance that students must actively seek may therefore invite the kind of agentic engagement that automatically delivered assistance does not. These distinctions underscore the importance of examining not only whether students receive AI feedback, but which psychological resources determine whether they engage with it productively.

Because agentic engagement and AI feedback literacy may appear to overlap at first reading, we draw an explicit conceptual boundary. Agentic engagement, in our usage, is a behavioral construct directed at learning agents, whether teachers, peers, or AI systems; it indexes whether students enter the learning activity as active contributors. AI feedback literacy is a competency construct directed at feedback events; it indexes the appraisal, judgment, affective management, and action capacities students bring to bear on AI-generated feedback. The two constructs share the dimension of doing something with feedback, but the agentic engagement scale items operationalize this as proactive contribution to the learning environment, whereas the AI feedback literacy items operationalize it as the cognitive-affective work of metabolizing a particular feedback episode. Self-determination theory is invoked throughout as a supplementary motivational lens that helps explain why the two constructs should be reciprocally supportive, but it is not the focal explanatory framework of the present study and is not directly operationalized.

### 2.3 The case for reciprocal within-person dynamics

Although AI feedback literacy and agentic engagement have evolved in separate literatures, their theoretical scaffolding suggests they should function as reciprocal psychological resources. Three lines of reasoning support this proposition. The first draws on self-determination theory. AI feedback literacy supports both competence need satisfaction, by giving students a sense that they can productively make use of AI feedback, and autonomy need satisfaction, by enabling them to evaluate and selectively act on feedback rather than uncritically accept it (Vansteenkiste et al., 2020). Need satisfaction in turn fuels the autonomous motivational orientation from which agentic engagement issues (Reeve and Tseng, 2011). Conversely, students who engage agentically with their learning environments accumulate the repeated, varied feedback encounters through which literacy is built; without such engagement, feedback literacy has nowhere to develop (Winstone et al., 2017a; Carless, 2019).

The second line draws on the mutualism principle in educational psychology, articulated most explicitly in Marsh and Craven's (2006) reciprocal effects model of self-concept and achievement, and recently revisited in motivation-achievement cycles by Vu et al. (2022). The mutualism principle holds that, in domains where psychological resources support one another developmentally, growth in one resource simultaneously enables and is enabled by growth in the other. Cross-sectional snapshots of correlated resources are therefore symptomatic of mutualism, but only longitudinal designs can distinguish mutualism from spurious co-occurrence (Burns et al., 2020).

Three boundary conditions distinguish the cases in which the mutualism principle applies from those in which it does not. First, the two resources must change at a similar time scale, so that their dynamic interplay can be observed within the study window. AI feedback literacy and agentic engagement are both expected to fluctuate at the within-semester scale, consistent with the six-week intervals used here. Second, the two resources must be supported by overlapping motivational processes, so that growth in one creates conditions favorable to growth in the other rather than depleting them. The self-determination-theoretic argument advanced above identifies competence and autonomy need satisfaction as the shared motivational substrate. Third, the two resources must be operationally distinct, so that their reciprocal estimates do not collapse into autocorrelation. The conceptual boundary articulated at the end of Section 2.2, together with the empirical separation of their measurement, satisfies this condition.

The third line draws on the specific affordances of AI feedback as a learning context. Because AI feedback is abundant and immediate, the relevant constraint on its use is not access but the learner’s capacity to interrogate, filter, and apply it. Where this capacity is high, students extract value from AI feedback by acting upon it iteratively, which is itself a form of agentic engagement directed at the AI as an interlocutor. Where this capacity is low, AI feedback risks becoming a substitute for student thinking rather than a scaffold for it. Recent work by Liu et al. (2024) on AI-mediated informal digital learning shows that motivation and enjoyment, both proximal indicators of need satisfaction, mediate the relationship between AI use and engaged learning behavior, supporting this conditional logic.

These three lines converge on a common prediction: that AI feedback literacy and agentic engagement co-develop reciprocally over time. They are not merely correlated; they should function as mutual catalysts within individuals. Importantly, this prediction is distinct from the weaker claim that students who are higher in AI feedback literacy on average are also higher in agentic engagement on average, which would describe a stable between-person association consistent with shared trait substrate. The reciprocal dynamics prediction is fundamentally a within-person claim: when a given student is, on a given occasion, above their own typical level of one resource, they should subsequently be above their own typical level of the other.

We note that the theoretical case for reciprocity articulated above is conceptually prior to, and distinct from, the statistical model used to test it. The RI-CLPM provides the analytic apparatus for evaluating whether the predicted within-person reciprocal patterns emerge in the data, but the evidential weight of the mutualism argument rests on the convergence of the self-determination-theoretic, mutualism-principle, and AI-affordance reasoning lines, not on the statistical model per se. The RI-CLPM is the test, not the theory.

### 2.4 Hypotheses

We specify three *a priori* expectations derived from the foregoing argument: two confirmatory within-person hypotheses (H2a and H2b) and one exploratory between-person research question (designated H1 below for ease of reference). The exploratory status of H1 reflects the fact that the precision with which between-person trait variance can be identified in a three-wave RI-CLPM depends on the size of that variance in the data and cannot be specified in advance. Figure 1 presents the conceptual model.

**Figure 1:**
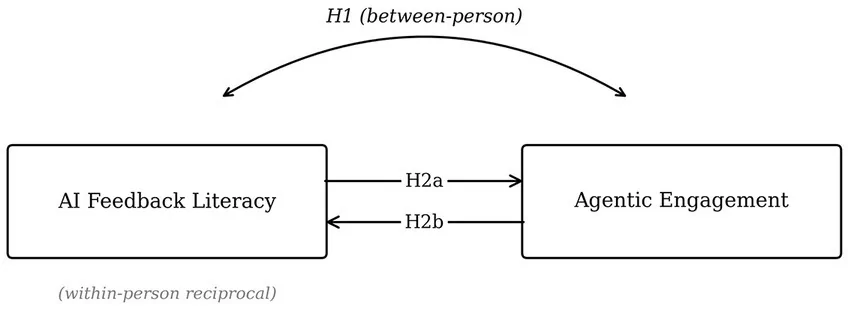
Conceptual hypothesized model of reciprocal dynamics between AI feedback literacy and agentic engagement. H1 = exploratory between-person trait-level association between AI feedback literacy and agentic engagement, evaluated through the correlation of the random intercepts. H2a and H2b = confirmatory within-person cross-lagged effects estimated across three measurement waves. The curved double-headed arrow represents the between-person correlation associated with H1; horizontal single-headed arrows represent the within-person reciprocal effects tested in H2a and H2b.

Hypothesis 1 (between-person association; exploratory). At the between-person level, students’ trait-like AI feedback literacy and trait-like agentic engagement may be positively associated, reflecting a shared psychological foundation predicted by self-determination theory. We treat this as an exploratory expectation rather than a confirmatory hypothesis: with three measurement waves and constructs that may be predominantly state-like in the present window, the random intercept variances and their covariance may be estimated only imprecisely, and the trait-level correlation may not be reliably distinguishable from zero. We therefore report the between-person estimates with appropriate caveats and do not anchor the substantive case for mutualism on the trait-level baseline. Within-person reciprocal dynamics, tested in H2a and H2b below, carry the primary theoretical weight of the study.

Hypothesis 2 (within-person reciprocal effects). At the within-person level, deviations in AI feedback literacy at wave t positively predict deviations in agentic engagement at wave t + 1 (H2a), and deviations in agentic engagement at wave t positively predict deviations in AI feedback literacy at wave t + 1 (H2b). These hypotheses operationalize the mutualism prediction: support for both H2a and H2b would indicate that the two constructs reciprocally enable each other after accounting for any stable between-person components captured by the random intercepts, regardless of the magnitude of those components.

Although both H2a and H2b specify positive cross-lagged effects, we do not hypothesize which of the two positive pathways is larger—that is, we make no directional prediction about asymmetry. The theoretical literature provides plausible arguments for either direction of asymmetry, and at present the empirical basis for adjudication is too thin to anchor a confirmatory prediction. We therefore report the comparison of the two cross-lagged paths as an exploratory test, with substantive interpretation deferred to the Discussion.

## 3 Method

### 3.1 Participants and procedure

Participants were 480 undergraduate students recruited from two comprehensive universities in eastern China. Inclusion criteria were enrollment in a non-English major and current undergraduate standing in the second or third year of study. Non-English majors were targeted to avoid the potential ceiling effects on AI feedback literacy that English-major students might display due to specialized training. The second and third years were chosen to balance two practical concerns: first-year students typically have limited exposure to AI-mediated coursework in the institutional context, whereas fourth-year students are subject to high attrition risk due to graduation and job-search activity. Recruitment followed a quota-stratified procedure designed to ensure balanced representation across sex, academic year, and disciplinary affiliation. Quotas were specified in advance to approximate the enrollment proportions at the two participating universities (sex: 55% women, 45% men; academic year: 55% second-year, 45% third-year; discipline: 25% humanities, 30% social sciences, 20% natural sciences, 25% engineering). Recruitment within each stratum proceeded on a first-come, first-included basis from a pool of volunteers who responded to recruitment notices in general-education courses, and continued until the stratum quota was filled. The integer-valued cell sizes shown in Table 1 are a direct consequence of this quota design rather than an artifact of random sampling. Baseline AI use frequency was not a quota dimension; it was measured at Wave 1 and is reported in Table 1 for descriptive purposes.

**Table 1:** Sample demographic characteristics (*N* = 480).

Characteristic	*n*	%
Sex		
Women	263	54.8
Men	217	45.2
Academic year		
Second year	271	56.5
Third year	209	43.5
Discipline		
Humanities	118	24.6
social sciences	141	29.4
natural sciences	109	22.7
Engineering	112	23.3
AI use frequency (baseline)		
1 (rarely)	14	2.9
2	67	14.0
3 (a few times per week)	178	37.1
4	165	34.4
5 (daily)	56	11.7

Percentages may not sum to 100 due to rounding.

The sample comprised 263 women (54.8%) and 217 men (45.2%), with 271 s-year (56.5%) and 209 third-year students (43.5%). Disciplinary distribution spanned humanities (24.6%), social sciences (29.4%), natural sciences (22.7%), and engineering (23.3%). At baseline, participants reported using AI tools for academic purposes with a median frequency of three to four times per week (5-point scale: 1 = rarely, 5 = daily). Detailed sample characteristics are shown in Table 1.

Data were collected at three measurement waves separated by six-week intervals across a single academic semester. The six-week interval was chosen to balance statistical power and substantive sensitivity: shorter intervals risk underestimating within-person change because measurement noise dominates the signal, whereas longer intervals introduce confounding from cross-semester transitions such as course changes and instructor changes (Vu et al., 2022). The selected interval is consistent with recent applications of RI-CLPM in motivation and engagement research (Granziera and Perera, 2019).

Beyond these methodological considerations, the six-week interval is theoretically grounded in the temporal grain at which AI feedback literacy and agentic engagement are expected to influence each other. Both constructs require repeated behavioral cycles—receiving AI feedback, appraising it, deciding whether to act, and observing the consequences—before measurable within-person change accrues. Intervals shorter than 4 weeks risk capturing measurement noise rather than genuine developmental fluctuation, because students may not have completed enough feedback-action cycles to shift their standing on either construct. Intervals longer than 8 weeks within a single semester risk introducing confounds from midterm transitions, assignment phase changes, and evolving familiarity with specific AI tools. The six-week spacing thus represents a theoretically motivated compromise: long enough for feedback-engagement cycles to accumulate, short enough to remain within a stable instructional context.

Of the 480 students who completed Wave 1, 437 (91.0%) completed Wave 2, and 401 (83.5%) completed Wave 3. Little’s MCAR test indicated that the missing data pattern was consistent with missing completely at random, χ^2^(38) = 41.83, *p* = 0.31. Given the high retention rate and MCAR-consistent missingness, we used the 399 participants with complete data across all three waves as the analytic sample. Each participant provided informed consent and received nominal compensation upon completing each wave. The study protocol was approved by the institutional ethics committee of Sias University.

### 3.2 Measures

#### 3.2.1 AI feedback literacy

AI feedback literacy was assessed with the AI Feedback Literacy Scale developed and validated by Liu and Deris (2025). The scale consists of 24 items organized into four subdimensions paralleling the Carless and Boud (2018) framework as adapted to AI feedback contexts: appreciating AI feedback (6 items; e.g., “I recognize that AI feedback can provide useful perspectives on my work”), judging AI feedback (6 items; e.g., “I can tell when AI feedback is reliable and when it is not”), managing affective responses to AI feedback (6 items; e.g., “I do not feel discouraged when AI feedback identifies many issues in my work”), and taking action on AI feedback (6 items; e.g., “After receiving AI feedback, I revise my work based on its suggestions”). Items were rated on a 5-point Likert scale ranging from 1 (strongly disagree) to 5 (strongly agree). The original validation reported Cronbach’s α coefficients ranging from 0.87 to 0.91 across subdimensions and a four-factor confirmatory factor analytic solution. In the present sample, a confirmatory factor analysis of the four-factor structure at Wave 1 yielded acceptable fit, χ^2^(246) = 412.71, *p* < 0.001, CFI = 0.968, TLI = 0.961, RMSEA = 0.042, SRMR = 0.038, and was significantly superior to a one-factor alternative, Δχ^2^(6) = 187.43, *p* < 0.001, confirming the dimensional structure in our sample. Cronbach’s α coefficients for the total scale were 0.89, 0.91, and 0.91 at Waves 1, 2, and 3 respectively; subdimension α coefficients ranged from 0.81 to 0.92 across waves. To maintain model parsimony and statistical power for the focal reciprocal question, the RI-CLPM was estimated on total composite scores rather than on a four-factor latent structure; a follow-up sensitivity analysis using a second-order factor model yielded substantively equivalent cross-lagged estimates (within ± 0.02 of those reported).

#### 3.2.2 Agentic engagement

Agentic engagement was assessed with the 10-item Agentic Engagement Scale developed by Reeve (2013) as an expansion of the original 5-item measure (Reeve and Tseng, 2011). Sample items include “I let my teacher know what I’m interested in,” “I offer suggestions about how to make the class better,” and “During class, I ask questions to help me learn.” Following the contextualization procedure used by Liu et al. (2024) for AI-mediated learning research, the stem of each item was adapted to refer to learning encounters more broadly, encompassing both teacher-student interactions and student-AI interactions. For example, the original item “During class, I ask questions to help me learn” was rendered as “During my learning activities, whether with my teacher or with an AI tool, I ask questions to help me learn.” Two of the 10 items required only the teacher-AI parallel insertion; the remaining eight retained their original wording with minimal contextual elaboration. The adaptation was reviewed by two bilingual educational psychologists for content validity, and a CFA on the adapted scale at Wave 1 confirmed a unidimensional structure, χ^2^(35) = 67.83, *p* = 0.001, CFI = 0.978, TLI = 0.972, RMSEA = 0.044, SRMR = 0.033, with factor loadings ranging from 0.61 to 0.82. Cronbach’s α coefficients in the present sample were 0.88, 0.90, and 0.89 at Waves 1, 2, and 3, respectively. Both scales were translated from English to Chinese following a standard forward-and-back-translation procedure. Two bilingual researchers independently translated the items, a third bilingual researcher back-translated them, and a fourth researcher (a content expert in feedback literacy research) reconciled discrepancies. Discrepancies arose for 7 of the 34 total items and were resolved by consensus among the four researchers. A pilot test with 42 undergraduates outside the main sample confirmed item clarity and item-total correlations above 0.40 for all items. Together with the Wave 1 confirmatory factor analyses and internal consistency estimates reported above and the longitudinal invariance tests reported in Section 4.2, these results support the appropriateness of the adapted measures for the present population.

#### 3.2.3 Control variables and marker variable

Sex (0 = women, 1 = men), academic year (2 = second year, 3 = third year), discipline (humanities, social sciences, natural sciences, engineering), and baseline AI use frequency were assessed at Wave 1. A five-item marker variable assessing attitudes toward an unrelated topic (campus cafeteria preferences) was included at each wave to enable a partial-correlation-based check for common method variance (Podsakoff et al., 2024).

### 3.3 Analytic strategy

Analyses proceeded in five stages. First, we computed descriptive statistics, internal consistency indices, and wave-by-wave Pearson correlations. Second, we examined longitudinal measurement invariance for each construct across the three waves following the procedure outlined by Putnick and Bornstein (2016), proceeding sequentially from configural to metric to scalar invariance, with partial scalar invariance acceptable if changes in CFI did not exceed 0.010 and changes in RMSEA did not exceed 0.015. Although the RI-CLPM was subsequently fit to composite scale scores rather than to latent factors with multiple indicators, measurement invariance results bear on the comparability of the composite means across waves and are reported here as a supplementary safeguard. Third, we tested for common method variance using Harman’s single-factor test as a preliminary screen and the marker variable approach as a more stringent secondary check (Podsakoff et al., 2024).

Prior to model estimation, two preparatory checks were performed. First, *a priori* power analysis using the powRICLPM R package (Mulder, 2023) indicated that, with three waves, *N* = 399 complete cases (the Wave 3 listwise sample, baseline *N* = 480), and conventional autoregressive paths (φ ≈ 0.30), the detectable cross-lagged effect at power = 0.80 and *α* = 0.05 was *β* = 0.13. Our observed cross-lagged estimates exceeded this threshold by a substantial margin. Second, Mardia’s multivariate skewness and kurtosis tests indicated significant departure from multivariate normality (skewness coefficient = 4.83, *p* = 0.002; kurtosis coefficient = 38.51, *p* < 0.001), justifying our choice of MLR over standard ML estimation. The first measurement wave was treated as the equilibrium state of the within-person process, following the recommendation of Mulder and Hamaker (2021); the Wave 1 within-person residual variances and their covariance were freely estimated rather than constrained to match the stationary distribution implied by the autoregressive and cross-lagged parameters.

Fourth, we fit the focal RI-CLPM. The model partitions the observed scores at each wave into between-person random intercepts and within-person residual components. Within-person residuals were regressed on their preceding within-person residuals (autoregressive paths) and on the preceding within-person residuals of the other variable (cross-lagged paths). Path coefficients were constrained to be equal across waves to test the stationarity assumption (Mulder and Hamaker, 2021). The cross-lagged path from AI feedback literacy to agentic engagement provided the test of H2a; the reverse path tested H2b; the covariance between the random intercepts provided the test of H1. Model fit was evaluated against established cutoffs: CFI ≥ 0.95, TLI ≥ 0.95, RMSEA ≤ 0.06, and SRMR ≤ 0.08 (Hu and Bentler, 1999). Maximum likelihood with robust standard errors (MLR) was used as the estimator.

Within-occasion associations between the AI feedback literacy and agentic engagement deviations were specified as freely estimated residual covariances at each wave. This is the standard RI-CLPM specification, and it treats contemporaneous associations as non-directional. The choice matters for interpretation. Speyer et al. (2025) show that when directional concurrent effects are present but modeled only as residual covariances, cross-lagged estimates can be biased, and Muthén and Asparouhov (2024) show that effects estimated as cross-lagged in such models may in fact be contemporaneous. Because a model with contemporaneous effects in both directions is not identified without instruments or additional constraints, we retained the standard specification and interpret the cross-lagged paths as prospective associations net of within-occasion covariation. We return to this point in the Discussion.

Fifth, we conducted three robustness analyses. We tested the equivalence of the two cross-lagged paths with a Wald test to evaluate possible asymmetry. We re-estimated a traditional CLPM omitting the random intercepts and compared its fit with the RI-CLPM using a likelihood-ratio test, providing a direct comparison of the two analytic approaches. Finally, we examined whether the cross-lagged effects survived inclusion of the covariates as predictors of the random intercepts. The optimal time-lag selection considerations of Dormann and Griffin (2015) informed the six-week wave spacing. All analyses were conducted in lavaan version 0.6.18 for R; data and analysis scripts will be deposited at the Open Science Framework upon publication.

## 4 Results

### 4.1 Sample characteristics and preliminary analyses

Sample demographic characteristics, including sex, academic year, disciplinary distribution, and baseline AI use frequency, are presented in Table 1. Means and standard deviations for AI feedback literacy and agentic engagement at each wave, along with Cronbach’s α coefficients and bivariate Pearson correlations across waves and constructs, are reported in Table 2. Both constructs exhibited moderate stability across the three waves, with wave-to-wave correlations ranging from 0.63 to 0.71 for AI feedback literacy and from 0.59 to 0.73 for agentic engagement. Concurrent correlations between the two constructs ranged from 0.22 to 0.33, indicating that the constructs are related but not redundant. Individual variables’ univariate skewness and kurtosis values fell within conventional thresholds (|skewness| < 2, |kurtosis| < 7); however, as reported in Section 3.3, Mardia’s test revealed significant multivariate non-normality, further justifying the use of MLR estimation.

**Table 2:** Descriptive statistics, internal consistency, and correlations of study variables across three waves.

Variable	*M*	SD	*α*	1	2	3	4	5	6
1. AIFL T1	3.24	0.81	0.876	—					
2. AIFL T2	3.36	0.83	0.877	0.709	—				
3. AIFL T3	3.49	0.85	0.887	0.629	0.711	—			
4. AE T1	3.53	0.80	0.882	0.219	0.321	0.284	—		
5. AE T2	3.54	0.77	0.871	0.266	0.330	0.327	0.648	—	
6. AE T3	3.61	0.82	0.892	0.210	0.336	0.324	0.588	0.725	—

AIFL, AI feedback literacy; AE, agentic engagement; T1, T2, T3 = Waves 1, 2, 3. All correlations *p* < 0.001.

### 4.2 Longitudinal measurement invariance

Measurement invariance testing across the three waves supported partial scalar invariance for AI feedback literacy and full scalar invariance for agentic engagement. Model fit indices for each invariance level are presented in Table 3. The drops in CFI and RMSEA from metric to scalar invariance fell within the conventional thresholds (ΔCFI < 0.010, ΔRMSEA < 0.015) for both constructs, with the exception of two item intercepts in the AI feedback literacy scale (one each in the appreciating and judging subdimensions) that were freed to vary across waves to achieve partial scalar invariance. Partial scalar invariance is sufficient for substantively meaningful longitudinal comparisons of construct means and reciprocal effects.

**Table 3:** Longitudinal measurement invariance tests for AI feedback literacy and agentic engagement.

Model	χ^2^	df	CFI	TLI	RMSEA	SRMR	ΔCFI	ΔRMSEA
AIFL: Configural	438.27	246	0.968	0.957	0.043	0.039	—	—
AIFL: Metric	467.83	266	0.964	0.955	0.044	0.044	−0.004	0.001
AIFL: Scalar (full)	561.49	286	0.947	0.938	0.051	0.051	−0.017	0.007
AIFL: Scalar (partial)	498.16	284	0.961	0.953	0.045	0.046	−0.003	0.002
AE: Configural	183.42	120	0.972	0.964	0.039	0.036	—	—
AE: Metric	198.71	129	0.969	0.962	0.041	0.039	−0.003	0.002
AE: Scalar (full)	217.83	138	0.964	0.958	0.043	0.041	−0.005	0.002

AIFL, AI feedback literacy; AE, agentic engagement. Partial scalar invariance for AIFL achieved by freeing two item intercepts across waves. All Δ values computed against the preceding less restrictive model.

Common method variance was assessed at two levels. Harman’s single-factor test indicated that a single factor accounted for 53.4% of the total variance, marginally above the conventional 50% threshold. However, this preliminary screen is widely recognized as insensitive to method bias (Podsakoff et al., 2024). The marker variable approach, a more stringent assessment, provided reassurance: partial correlations between AI feedback literacy and agentic engagement controlling for the marker variable changed by less than 0.02 from zero-order correlations, suggesting that common method variance did not meaningfully inflate the focal associations (Podsakoff et al., 2024). Moreover, the RI-CLPM partials out time-invariant method bias as part of the random intercept variance, providing an additional analytic safeguard (Hamaker et al., 2015).

### 4.3 Random-intercept cross-lagged panel model

The RI-CLPM provided excellent fit to the data, χ^2^(8) = 12.08, *p* = 0.148, CFI = 0.996, TLI = 0.993, RMSEA = 0.036, 90% CI [0.000, 0.074], SRMR = 0.019. We compared the focal model, in which autoregressive and cross-lagged paths were constrained equal across waves, against a freely estimated model in which these paths varied across waves. The unconstrained model returned χ^2^(4) = 7.96, *p* = 0.094, CFI = 0.997, RMSEA = 0.034, SRMR = 0.018. The constrained focal model showed no significant deterioration in fit relative to this unconstrained alternative, Δχ^2^(4) = 4.12, *p* = 0.390, ΔCFI = 0.001, supporting the stationarity assumption and the imposition of equality constraints. The model is depicted graphically in Figure 2, with all standardized path coefficients displayed.

**Figure 2:**
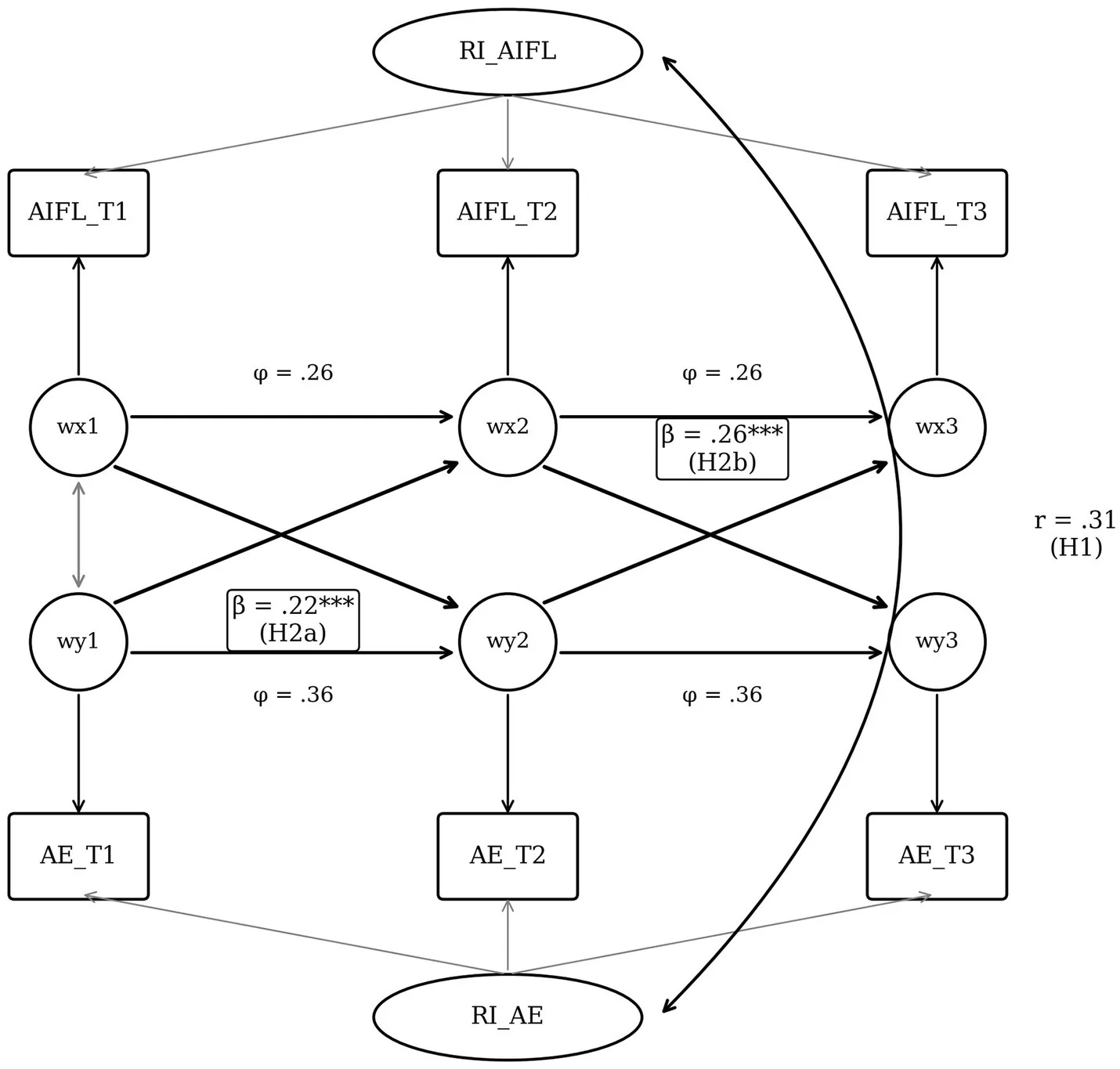
Random-intercept cross-lagged panel model with standardized estimates. AIFL_T1, AIFL_T2, AIFL_T3 = observed AI feedback literacy scores at Waves 1, 2, and 3; AE_T1, AE_T2, AE_T3 = observed agentic engagement scores at Waves 1, 2, and 3; wx1, wx2, wx3 = within-person deviations for AIFL; wy1, wy2, wy3 = within-person deviations for AE; RI_AIFL, RI_AE = random intercepts (between-person components). φ = autoregressive paths; *β* = cross-lagged paths. All cross-lagged and autoregressive paths are constrained to be equal across waves under the stationarity assumption. *** *p* < 0.001.

At the between-person level, both random intercept variances were sizeable and precisely estimated, with 95% confidence intervals well above zero, Var[RI_AIFL] = 0.394, 95% CI [0.301, 0.488], *p* < 0.001; Var[RI_AE] = 0.293, 95% CI [0.186, 0.399], *p* < 0.001. The correlation between random intercepts was *r* = 0.315, SE = 0.088, 95% CI [0.142, 0.487], *z* = 3.57, *p* < 0.001, supporting Hypothesis 1. The between-person component of the model indicates that students who scored systematically higher in AI feedback literacy across the semester also tended to score systematically higher in agentic engagement, consistent with a stable trait-level co-variation between the two constructs. This between-person association is conceptually distinct from the within-person reciprocal dynamics reported below.

At the within-person level, both cross-lagged paths were positive and significant, supporting Hypotheses 2a and 2b. Complete path coefficient estimates are presented in Table 4. Deviations in AI feedback literacy at a given wave positively predicted deviations in agentic engagement at the next wave, standardized *β* = 0.222, unstandardized *B* = 0.246, SE = 0.066, 95% CI [0.116, 0.376], *p* < 0.001. The reverse path, from agentic engagement to AI feedback literacy, was also positive and significant, standardized *β* = 0.263, unstandardized *B* = 0.249, SE = 0.060, 95% CI [0.131, 0.366], *p* < 0.001. The autoregressive paths were also positive and significant, with standardized φ_AIFL = 0.261 and standardized φ_AE = 0.358, indicating moderate within-person stability across the six-week intervals.

**Table 4:** Random-intercept cross-lagged panel model estimates.

Parameter	*B*	SE	*β*	95% CI	*z*	*p*
Between-person (random intercepts)						
Var(RI_AIFL)	0.394	0.048	—	[0.301, 0.488]	8.26	< 0.001
Var(RI_AE)	0.293	0.054	—	[0.186, 0.399]	5.40	< 0.001
r(RI_AIFL, RI_AE)	0.315	0.088	0.404	[0.248, 0.561]	3.57	< 0.001
Within-person autoregressive						
φ_AIFL	0.286	0.079	0.261	[0.132, 0.441]	3.63	< 0.001
φ_AE	0.342	0.094	0.358	[0.158, 0.525]	3.65	< 0.001
Within-person cross-lagged						
AIFL → AE (H2a)	0.246	0.066	0.222	[0.116, 0.376]	3.72	< 0.001
AE → AIFL (H2b)	0.249	0.060	0.263	[0.131, 0.366]	4.16	< 0.001

AIFL, AI feedback literacy; AE, agentic engagement; RI, random intercept; B, unstandardized estimate; *β*, standardized estimate; CI, confidence interval. H2a and H2b refer to within-person reciprocal hypotheses. Standardized estimates are reported on the wave-1 within-person scale. All cross-lagged paths are constrained to be equal across waves under the stationarity assumption.

### 4.4 Robustness checks and model comparison

To evaluate the contribution of separating between- and within-person variance, we compared the RI-CLPM against a traditional CLPM in which the random intercepts were omitted. The traditional CLPM provided substantially poorer fit, χ^2^(11) = 61.45, *p* < 0.001. A likelihood-ratio test indicated that the RI-CLPM was the better-fitting model, Δχ^2^(3) = 49.37, *p* < 0.001. Notably, the cross-lagged estimates in the traditional CLPM were substantially smaller than the corresponding RI-CLPM within-person estimates (CLPM: *β* = 0.116 for AI feedback literacy → agentic engagement, *β* = 0.157 for the reverse path; RI-CLPM: *β* = 0.222 and *β* = 0.263 respectively), indicating that the trait-level covariation captured by the random intercepts was suppressing rather than inflating the apparent within-person reciprocal dynamics in the traditional model—a pattern consistent with the known bidirectional bias of the CLPM (Lucas, 2023). Model comparison statistics are presented in Table 5, alongside the Wald test of cross-lagged path equivalence used to evaluate possible asymmetry. The Wald test was non-significant, indicating that the two reciprocal pathways did not differ in magnitude. The reciprocal dynamics, in other words, were symmetric. This pattern is visualized in Figure 3, which juxtaposes the within-person reciprocal estimates from the RI-CLPM against the corresponding estimates from the traditional CLPM.

**Table 5:** Model comparison: traditional CLPM vs. RI-CLPM and test of reciprocal symmetry.

Model / Test	χ^2^	df	*p*	CFI	RMSEA	SRMR	Δχ^2^	Δdf
Traditional CLPM (no RI)	61.45	11	< 0.001	0.957	0.107	—	—	—
RI-CLPM (focal model)	12.08	8	0.148	0.996	0.036	0.019	—	—
LR test: RI-CLPM vs. CLPM	—	—	< 0.001	—	—	—	49.37	3
Wald test: B(AIFL→AE) = B(AE → AIFL)	0.001	1	0.972	—	—	—	—	—

CLPM, cross-lagged panel model; RI-CLPM, random-intercept cross-lagged panel model; LR, likelihood ratio. The Wald χ^2^ statistic tests whether the two cross-lagged paths are statistically equivalent in magnitude. A non-significant Wald test supports the interpretation of symmetric reciprocity.

**Figure 3:**
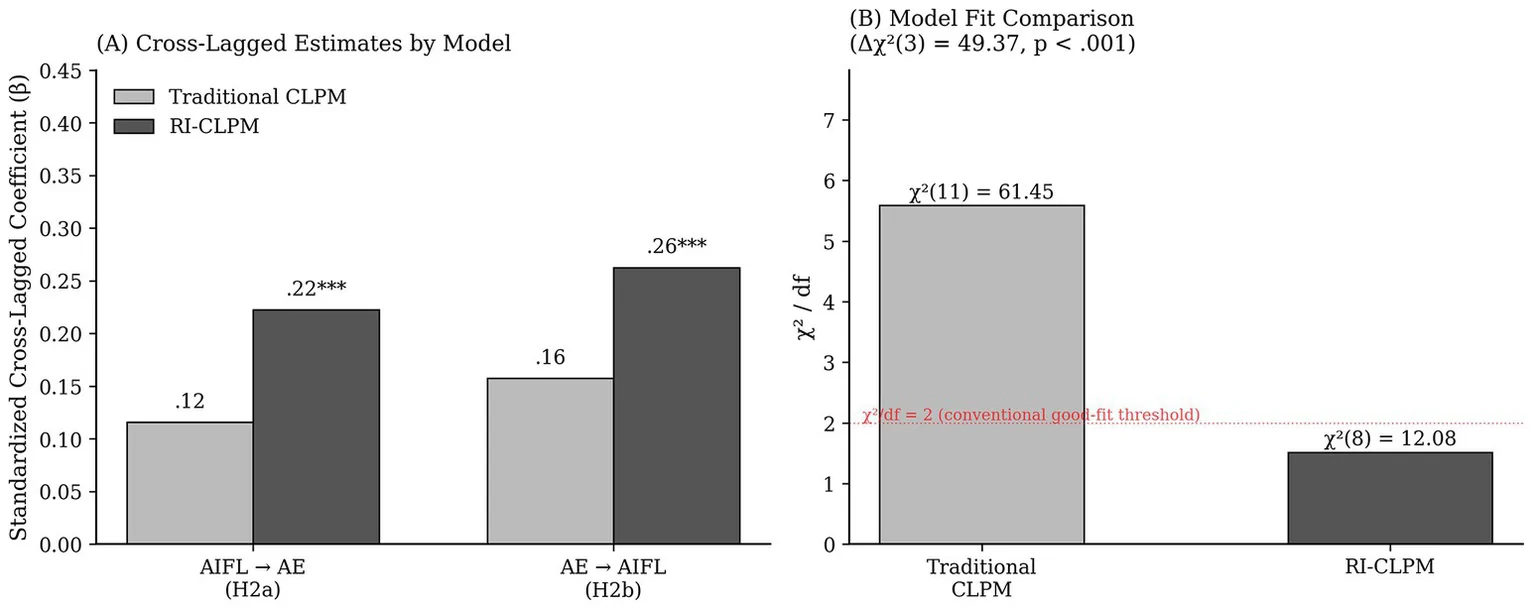
Comparison between the traditional cross-lagged panel model and the random-intercept cross-lagged panel model. Panel **A** presents standardized cross-lagged coefficients from the traditional CLPM (which omits random intercepts) and from the RI-CLPM. Panel **B** presents the χ^2^/df ratio for each model and the likelihood-ratio test of nested model comparison. The RI-CLPM yields larger within-person reciprocal estimates and a substantially better fit than the traditional CLPM, illustrating that the latter conflates between-person trait covariation with within-person reciprocal dynamics. *** *p* < 0.001.

Finally, we examined whether including sex, academic year, discipline, and baseline AI use frequency as predictors of the random intercepts altered the focal within-person estimates. The cross-lagged paths changed by less than 0.01 in standardized magnitude with covariates included, and their statistical significance was unaffected. Baseline AI use frequency was a positive predictor of both random intercepts (*β* = 0.194 for AI feedback literacy, *p* < 0.001; *β* = 0.128 for agentic engagement, *p* = 0.017). Sex showed a small positive association with the random intercept of AI feedback literacy (men slightly higher, *β* = 0.093, *p* = 0.064) but not with agentic engagement.

## 5 Discussion

This study set out to test whether AI feedback literacy and agentic engagement function as reciprocal psychological resources for Chinese undergraduates, using a three-wave RI-CLPM to separate trait-level covariation from within-person reciprocal dynamics. Three findings warrant emphasis. First, with respect to the exploratory between-person research question (H1), the random intercept variances of both constructs were sizeable and precisely estimated, and the trait-level correlation between AI feedback literacy and agentic engagement was moderate, positive, and statistically significant (*r* = 0.315, 95% CI [0.142, 0.487], *p* < 0.001), providing supportive evidence for H1 even under the conservative three-wave design. This finding indicates a robust stable trait-level association. Second, the two constructs reciprocally fueled each other at the within-person level, with both cross-lagged paths reaching conventional significance and both confidence intervals excluding zero. Third, the magnitudes of the two reciprocal pathways did not differ from each other, indicating a symmetric mutualism rather than a directional dependence. Taken together, the findings reposition AI feedback literacy from a static competence to a dynamic developmental resource, and they provide longitudinal evidence for what cross-sectional studies have suggested only correlationally.

### 5.1 Theoretical implications

The findings extend student feedback literacy theory in three respects. First, they push the construct from a primarily cognitive-evaluative competency, as articulated in the Carless and Boud (2018) framework, into a developmental resource embedded in students’ broader agentic stance toward learning. The strong within-person path from agentic engagement to subsequent AI feedback literacy indicates that engagement is not merely a downstream consequence of having developed literacy but is part of the very process by which literacy accumulates. Students who proactively ask questions of AI feedback systems, formulate follow-up prompts, and contest or revise the outputs they receive are doing the work through which appraisal, judgment, and action capacities are formed. This is consistent with Carless's (2019) characterization of feedback as a spiral process and with Chong's (2021) ecological framing, but it gives those framings empirical traction in the longitudinal sense that the RI-CLPM design permits.

Second, the findings sharpen the AI feedback literacy construct against alternative accounts. One alternative interpretation of cross-sectional findings in this area is that AI feedback literacy is a derivative of more general academic engagement, with no distinctive psychological structure. The present within-person results refute this interpretation: deviations in AI feedback literacy contain unique predictive information about subsequent deviations in agentic engagement, after partialling between-person variance captured by the random intercepts. AI feedback literacy is therefore not a verbal proxy for engagement but a partially distinct developmental resource that engagement helps build and that, in turn, helps build engagement.

Third, the symmetric mutualism finding has implications for theorizing the directionality of feedback processes. The traditional feedback literacy literature has been ambivalent on directionality, sometimes treating literacy as the precursor to productive uptake and other times treating uptake as a way of building literacy. The present results suggest that this ambivalence reflects something real in the phenomenon itself: there is no privileged temporal direction at the within-person level. Each construct both predicts and is predicted by the other over the time scales studied here. This pattern matches the mutualism account most closely associated with the reciprocal effects model in academic self-concept research (Marsh and Craven, 2006) and the motivation-achievement cycles articulated by Vu et al. (2022). Vu et al. (2022) reported symmetric reciprocity between motivation and achievement in a meta-analytic synthesis, and they interpreted that symmetry as evidence that the two constructs were sustained by overlapping motivational mechanisms rather than by directional dependency. Our finding extends their pattern from a single-person-internal cognitive-motivational coupling to a coupling between a person-internal competency (AI feedback literacy) and a person-environment behavioral interface (agentic engagement). The implication is that mutualism is not confined to dyads of psychological constructs sharing a common motivational substrate; it can also emerge when one node of the reciprocal pair is itself a behavior directed outward, provided that the behavior systematically generates conditions favorable to the other node’s growth. This generalization broadens the empirical reach of mutualism as a theoretical principle and pushes against unidirectional intervention logics that assume training feedback literacy will straightforwardly produce more engaged learners, or that engaging learners will straightforwardly produce more literate ones.

An important caveat is that temporal precedence in a cross-lagged model does not, by itself, establish causation. The RI-CLPM controls for stable between-person confounds via the random intercepts and for prior levels of each construct via the autoregressive paths, but it cannot rule out time-varying third variables that simultaneously drive both AI feedback literacy and agentic engagement across waves. The present findings are therefore best interpreted as evidence that the two constructs track each other’s within-person fluctuations in a manner consistent with the mutualism hypothesis, rather than as definitive proof of bidirectional causation. The same caution applies to the lag structure: effects estimated as cross-lagged may partly reflect contemporaneous influence (Muthén and Asparouhov, 2024; Speyer et al., 2025), and with a six-week lag we cannot exclude that some of the mutual influence between the two constructs unfolds within rather than across measurement occasions.

A related consideration concerns the time scale of the constructs under study. Feedback literacy is typically conceptualized as a competence that accumulates over extended developmental periods, akin to a cognitive schema. If it fluctuates detectably within six-week windows, as our within-person paths suggest, the question arises whether the RI-CLPM is capturing genuine literacy development or merely short-term shifts in cognitive strategy activation—a kind of “feedback confidence” that rises and falls with recent usage intensity. We suggest that both processes are likely at work: the within-person reciprocal spiral documented here may represent the short-term mechanism through which longer-term literacy eventually consolidates, much as daily practice episodes in skill acquisition accumulate into stable expertise. Whether the short-term within-person oscillations observed here do in fact sediment into the stable between-person trait captured by the random intercepts is a question that multi-year longitudinal designs are needed to answer.

The findings also speak to self-determination theory. SDT predicts that competence and autonomy need satisfaction together support the autonomous motivational orientation underlying agentic engagement (Vansteenkiste et al., 2020). AI feedback literacy can be read as one specific channel through which both competence (the sense that one can productively make use of AI feedback) and autonomy (evaluating and selectively acting on AI feedback rather than uncritically accepting it) are realized in AI-rich learning contexts. The reciprocal pattern observed here is consistent with the SDT-derived expectation that need satisfaction and need-satisfying behavior co-develop in a virtuous spiral.

The behavioral dynamics observed here also connect to emerging models of how interaction patterns with large language models shape learning-related behavior more broadly. Jyothy et al. (2024) applied the Fogg Behavior Model to students’, teachers’, and researchers’ use of LLMs and found that students’ and researchers’ use of these tools was shaped chiefly by their motivation and ability. The model holds that a behavior occurs when motivation, ability, and a trigger converge—three elements that map onto the competence satisfaction (ability), autonomous motivation (motivation), and feedback event (trigger) triad implicit in our theoretical argument. The reciprocal mutualism we documented may thus reflect a self-reinforcing alignment of these three behavioral prerequisites.

### 5.2 Methodological implications

The substantial improvement in fit from CLPM to RI-CLPM, alongside the reduction in cross-lagged estimates when trait variance was not partialled out, carries an important methodological message for feedback research more broadly. Studies that have used CLPM to estimate reciprocal effects between feedback-related constructs and engagement-related constructs have likely produced estimates that are biased upward in some respects and downward in others, depending on the relative magnitude of trait covariation. In our data, the RI-CLPM within-person cross-lagged effects were approximately 1.7 to 1.9 times larger than the corresponding CLPM estimates, indicating that the traditional model’s conflation of between- and within-person variance suppressed rather than inflated the reciprocal signal. The direction of this bias is not knowable in advance: depending on the relative magnitudes of trait covariance and within-person dynamics, the traditional CLPM can either inflate or deflate the within-person estimates of interest (Lucas, 2023). The RI-CLPM should therefore become the default analytic strategy for reciprocity questions in this field. Its three-wave version, applied here, represents the minimal sufficient design for identification (Mulder and Hamaker, 2021); four or more waves would permit stronger tests, including relaxing the stationarity assumption and modeling time-varying effects, and we recommend such designs for confirmatory follow-up work.

A second methodological note concerns the partial scalar invariance encountered in the AI feedback literacy scale across waves. Two item intercepts shifted slightly across waves, an unsurprising finding given the rapid evolution of student exposure to AI tools during the data collection window. The literature is converging on the position that partial scalar invariance is sufficient for meaningful longitudinal comparisons when the freed intercepts are few and substantively interpretable (Putnick and Bornstein, 2016). Future work using newer AI feedback literacy scales should anticipate similar item-level drift and design analyses accordingly.

### 5.3 Practical implications

Two practical implications follow from the reciprocal mutualism we have documented. First, interventions aimed at developing students’ productive use of AI feedback should not treat AI feedback literacy and agentic engagement as separable training targets. A program that teaches students how to evaluate and act on AI feedback in the abstract, without simultaneously creating the conditions in which they exercise agency, leaves half the developmental loop ungrounded. Conversely, encouraging students to engage agentically with AI tools without scaffolding their evaluation skills risks producing a kind of confident misuse that may be worse than passive avoidance. Curriculum designers should plan integrated experiences that exercise both resources in sequence and in interaction.

Second, the symmetric mutualism finding implies that intervention entry points are flexible. Educators can productively begin from either resource, whichever is more accessible to mobilize in a given pedagogical context. A first-year writing seminar might enter through agentic engagement scaffolds, asking students to formulate questions and contest AI suggestions, with literacy building as a byproduct of repeated engagement. A senior research methods course might enter through literacy training, asking students to systematically evaluate AI-generated summaries of empirical studies, with engagement building as competence accumulates. Both entry points should be viable in principle. However, we counsel caution against treating the two resources as fully interchangeable starting points in all contexts. Because one core pillar of AI feedback literacy is the capacity to judge when AI output is unreliable, encouraging agentic engagement with AI tools before students possess a baseline of critical appraisal skill risks producing confident misuse—students who interact energetically with AI feedback yet uncritically internalize its errors. In the initial phases of students’ exposure to AI feedback (e.g., first-year undergraduates encountering AI writing tools for the first time), educators may therefore need to prioritize the literacy entry point, scaffolding critical evaluation before inviting open-ended agentic exploration. Once a minimum evaluative threshold is crossed, the symmetric mutualism documented here should sustain itself with less direct instructional intervention.

Beyond these structural recommendations, three concrete instructional heuristics follow directly from our findings. First, instructors can require students to prepare a brief AI-interrogation protocol before each major assignment, specifying the kinds of questions they intend to ask the AI, the criteria by which they will evaluate its responses, and the conditions under which they will refuse a suggestion. This protocol externalizes the appraisal and judgment dimensions of AI feedback literacy and creates a structured occasion for agentic engagement at the planning stage. Second, students can maintain a feedback log in which they record each substantive AI feedback episode, their evaluation of its quality, and the action they took in response. The act of logging consolidates the take-action dimension of literacy while documenting the feedback-related agency that the RI-CLPM identified as a within-person catalyst. Third, periodic in-class “feedback critique” sessions can be used to surface variability in how students appraise the same AI output, exposing them to the reasoning of peers and creating discursive opportunities for both literacy and agency to develop in parallel. Each of these heuristics targets the reciprocal loop rather than either construct in isolation, consistent with the mutualism logic of our findings.

### 5.4 Limitations and future directions

Several limitations bound the present conclusions. The sample, drawn from two Chinese universities and restricted to second- and third-year non-English majors, may not generalize to other educational systems, age groups, or disciplinary contexts. We note, however, that the Chinese higher education context is not simply a generalization constraint but is theoretically informative. Within Confucian Heritage Culture, agentic engagement has historically been characterized as culturally muted, with students socialized to defer to authoritative knowledge sources and to display agency in less visible, more deliberative ways than is typical in Western educational settings (Cheon et al., 2019). The fact that we nonetheless observed robust within-person reciprocal effects between AI feedback literacy and agentic engagement is noteworthy. One speculative interpretation—which the present data cannot verify—is that AI feedback may offer a less culturally-loaded venue in which Chinese students can exercise agency, free of the social risks attached to questioning or contesting a human authority. This conjecture, advanced here strictly as a hypothesis for future investigation rather than as a conclusion supported by the present data, invites cross-cultural work that could test directly: in cultural contexts where teacher-directed agency carries higher social cost, AI-mediated reciprocal dynamics may be especially pronounced, whereas in contexts where teacher-directed agency is normalized, the reciprocal pattern observed here may merge with, rather than supplement, the reciprocal dynamics of teacher-directed engagement. Comparative cross-cultural work would help establish the boundary conditions of the reciprocal pattern we observed and would adjudicate this conjecture.

Relatedly, the present findings should not be generalized uncritically across AI systems, academic disciplines, or feedback contexts. Our measures referred to AI feedback and AI tools in general terms, without distinguishing among AI systems or feedback tasks, and our participants were non-English majors from four broad disciplinary groups; whether the same reciprocal pattern holds for particular combinations of tool and task, such as AI feedback on STEM problem-solving, creative work, or clinical training, remains an open question. Different AI systems vary in feedback specificity, reliability, and interactional affordance, and these differences may moderate the strength or even the direction of the reciprocal dynamics documented here.

The reliance on self-report measures for both constructs raises the possibility of common method variance, although the marker variable analysis and the RI-CLPM’s partialling of time-invariant method bias mitigate this concern substantially. Future work could triangulate self-reports with behavioral indicators, such as actual interaction patterns with AI feedback tools captured through log data (Darvishi et al., 2024), or with instructor ratings of agentic engagement. A more fundamental limitation is that self-reports capture perceived rather than enacted feedback use: students may overestimate how critically they evaluate AI output, and reports of agentic behavior may reflect intentions more than conduct. Behavioral indicators, such as prompt and revision histories from AI writing tools or the proportion of AI suggestions that students accept, modify, or reject across drafts, would allow future studies to test whether the reciprocal pattern also holds for observed feedback-use behavior.

The three-wave design represents the minimal sufficient configuration for RI-CLPM identification, and although the stationarity constraints fit the data adequately here, four or more waves would permit relaxation of these constraints and tests of whether the reciprocal dynamics intensify, attenuate, or remain stable across longer time spans. The six-week interval was selected for substantive reasons, but the question of optimal time lag for within-person feedback dynamics remains open and merits systematic investigation, following the time-lag sensitivity logic articulated by Dormann and Griffin (2015).

The changing landscape of AI tool availability during the data collection period represents a further limitation. Students’ frequency, type, and purpose of AI use may have shifted systematically across the three waves as new tools became available or as familiarity with existing tools deepened. Such shifts constitute time-varying confounds that the RI-CLPM does not explicitly model. Future designs could incorporate wave-level AI usage logs as time-varying covariates to disentangle the reciprocal dynamics of interest from the effects of escalating tool exposure.

In addition, the observed within-person associations admit alternative longitudinal interpretations beyond the mutual-development mechanism we have foregrounded. The cross-lagged patterns could partly reflect stable developmental trends (e.g., both constructs increasing monotonically as students mature across the semester) or unmeasured time-varying influences (e.g., assignment difficulty, exam proximity) that simultaneously shift both constructs. Although the RI-CLPM’s partitioning of trait variance mitigates the first concern, the second remains a limitation inherent to observational panel designs. Experimental or quasi-experimental manipulations of AI feedback literacy training, combined with longitudinal measurement, would provide stronger causal leverage.

Finally, we did not measure boundary conditions that may moderate the reciprocal dynamics. Candidates for moderating variables include the autonomy-supportiveness of instructional contexts, the type and quality of AI tools accessed (general-purpose chatbots vs. domain-specific feedback systems), and learners’ baseline self-efficacy beliefs. Future work could productively examine these moderators using extensions of the RI-CLPM that incorporate latent moderation or multi-group structures.

Notwithstanding these limitations, the present study provides what we believe is the first longitudinal evidence that AI feedback literacy and agentic engagement co-develop reciprocally at the within-person level among university students. The symmetric mutualism we documented invites a reframing of AI feedback literacy in educational psychology: not as a static competence to be installed in students through training, but as a developmental resource that grows through the very engagement it makes possible.

## 6 Conclusion

Whether students grow into autonomous learners under the conditions of pervasive AI feedback depends on a set of psychological resources that we have shown to be reciprocally entangled rather than independently distributed. AI feedback literacy and agentic engagement, two constructs that have traveled in separate literatures, turn out to be reciprocally linked within individual students. The present three-wave design did afford a precise and significant estimate of a stable trait-level association between the two constructs, and the data additionally showed that they reciprocally predicted each other across the semester at the within-person level, with neither serving as the privileged precursor of the other.

At the empirical level, the three-wave data directly support three conclusions: (a) the two constructs share a stable between-person association, (b) they exhibit significant positive within-person cross-lagged effects in both directions after trait variance is partialled out, and (c) the two cross-lagged effects did not differ significantly in magnitude. The broader theoretical claim—that AI feedback literacy is not a static property students bring to learning environments but a developmental resource that grows through engaged use—is an interpretation that the data are consistent with, but that requires confirmation through longer time spans, experimental manipulation, and cross-cultural replication. The findings also commit feedback literacy theory to a longitudinal stance that earlier cross-sectional research could only gesture toward. As universities continue to integrate AI tools into their curricula at scale, the practical task is no longer to train students to use AI feedback well in the abstract. It is to design learning environments in which the reciprocal mutualism we have documented can take hold.

## Data Availability

The raw data supporting the conclusions of this article will be made available by the authors, without undue reservation.
